# Demographic Analysis of Emergency Department Patients at the Ruijin Hospital, Shanghai

**DOI:** 10.1155/2011/748274

**Published:** 2011-07-06

**Authors:** Wim Lammers, Willem Folmer, Esther M. M. Van Lieshout, Terry Mulligan, Jan C. Christiaanse, Dennis Den Hartog, Jianjing Tong, Yiming Lu, Peter Patka

**Affiliations:** ^1^Department of Surgery-Traumatology, Erasmus MC, University Medical Center Rotterdam, P.O. Box 2040, 3000 CA Rotterdam, The Netherlands; ^2^Department of Emergency Medicine, Erasmus MC, University Medical Center Rotterdam, P.O. Box 2040, 3000 CA Rotterdam, The Netherlands; ^3^Safety Region Rotterdam Area, P.O. Box 9154, 3007 AD Rotterdam, The Netherlands; ^4^Department of Emergency Medicine and Acute Surgery-Traumatology, Ruijin Hospital, Shanghai Jiao Tong University School of Medicine, No. 197 Rui Jin Er Road, Shanghai 20025, China

## Abstract

Emergency medicine is an upcoming discipline that is still under development in many countries. Therefore, it is important to gain insight into the organization and patients presenting to the Emergency Department (ED). The aim of this cross-sectional study was to provide an epidemiological description of complaints and referrals of the patients visiting the ED of the Ruijin Hospital in Shanghai, China. A questionnaire was developed and completed for a convenience sample of all patients presenting to the Triage Desk of the ED. The study was performed in June 2008. A total of 2183 questionnaires were completed. The most common complaints were fever (15%), stomach/abdominal pain (15%), vertigo/dizziness (11%), and cough (10%). Following triage, patients were predominantly referred to an internist (41%), neurologist (14%), pulmonologist (11%), or general surgeon (9%). This study provides a better understanding of the reason for the ED visit and the triage system at the ED of the Ruijin Hospital. The results can be used in order to improve facilities appropriate for the specific population in the ED.

## 1. Introduction

Emergency medicine (EM) is an evolving discipline in China as the economy modernization and urbanization are progressing rapidly. Consequently, there is an increasing need for a well-organized healthcare system that keeps up with the changing needs [1]. Shanghai is one of the largest Chinese cities, where a major public event, the World Exposition, was organized in 2010.

In order to estimate EM organization, a cooperative project between the Ruijin Hospital in Shanghai (China) and the Erasmus MC (Rotterdam, The Netherlands) was initiated. The primary aim of this collaboration is to perform research into the current situation and development of care provided at the Emergency Department (ED). An early step for the development and modernization of any ED is an assessment of needs, which will define areas of attention for improvements [2]. Assessing the organizational structure, including the type of patients and their referral, is an important start. This very much applies to EM because it is one of the few specialized areas of medicine that is determined by the types of presentations of the patients [3].

At the Ruijin Hospital, emergency care is organized following a multidisciplinary model that is also widely applied in Europe [4, 5]. The ED is managed by doctors from different disciplines; in addition to emergency physicians, different specialists are present at the ED at all time for consultation. The ED of the Ruijin Hospital consists of a corridor with rooms for each of the following areas of specialization: internal medicine, neurology, pulmonology, general surgery, neurosurgery, urology, cardiothoracic surgery, orthopedic surgery, obstetrics/gynecology, traditional Chinese medicine, stomatology, ENT (ear, nose, and throat diseases), dermatology, and ophthalmology. An independent critical care service, called the “Green pass way,” is also available. It is staffed by emergency physicians who treat the critically ill patients. These critically ill patients are admitted directly to the critical care area or/and the resuscitation room, which has its own entrance and sixteen monitored beds for providing acute critical care support. Separate, solitary Emergency Departments are available for pediatric and burn patients.

All patients, either walk-in (i.e., self-referral) or transported by ambulance, are first registered at the triage desk. The triage nurses subsequently refer them to one of the physicians. Before being seen by a physician, patients or their accompanying family should register the patient at the registration area where the consultation fee should be paid. The triage nurses refer patients to the appropriate physician for consultation, examination, and treatment. Any necessary complementary diagnostic examinations or laboratory tests need to be paid before being performed except for the critically ill patients. All diagnostic facilities for performing radiography, ultrasonography, computed tomography, electro cardiography, and laboratory tests are available 24 hours a day within the ED.

Every hospital department of the Ruijin Hospital, including the ED, has its own pharmacy where patients should obtain and pay for their prescribed medication upon discharge from the hospital. Depending upon the nature and severity of complaints or injuries, patients are either admitted to the ED or to one of the hospital departments of the consulting physicians. In addition to a general and trauma ward, the ED also has its own observation unit and ICU.

Until now, no demographic description of patients visiting the ED of the Ruijin Hospital has been published in the international literature [1]. Knowing how many patients visit the ED, what complaints they present with, and their referrals will provide insight into the current status of emergency care at the ED of the Ruijin Hospital in Shanghai. Therefore, the aim of this study was to perform a cross-sectional descriptive survey of patients visiting the ED of the Ruijin Hospital.

## 2. Materials and Methods

The current study was performed from June 5 to June 27, 2008, at the ED of the Ruijin Hospital (Shanghai, China). The Ruijin Hospital is the largest teaching hospital in Shanghai with 1800 beds and is affiliated to Shanghai Jiao Tong University School of Medicine. Data were collected for all patients referred to the triage desk at the ED. Patients reporting directly to the pediatric ED or the resuscitation room as well as patients visiting the ED for routine antibiotic infusion were not included.

A questionnaire was developed in order to collect data. The triage desk is staffed by nurses. Based upon their experience, these nurses refer the patients. There is currently no written protocol or formal triage system. At the Triage Desk the following information of the questionnaire were recorded: gender, date of birth, race (i.e., Asian or Non-Asian), date of visit, time of visit, type of transportation to the hospital (i.e., ambulance or self-referred), chief complaints (separated into 21 categories), and which specialist the patient was referred to (with the choice of 11 types of specialists and a free text field for options not listed). The triage nurses completed the questionnaires during office hours on Monday to Friday. 

The questionnaire was developed after extensive discussion with local specialists. Subsequently, five medical specialists and four medical students translated the questionnaire into Chinese. Another group of medical specialists verified the translation and made changes when necessary. This process was repeated until the translation was considered fully sufficient. The final questionnaire was bilingual, combining both the English and Chinese text.

Data were entered into a database and analyzed using the Statistical Package for the Social Sciences version 16.0 (SPSS, Chicago, Ill, USA). Frequencies were calculated for all items of the questionnaire. Age was presented as median with the 1st and 3rd quartile. Eight questionnaires that lacked details on complaints and consulting specialist were excluded from the analysis.

## 3. Results

A total of 2183 questionnaires were completed at the Triage Desk. Demographic data for this population are given in Table 1. The median age was 52 years (P_25_–P_75_ 31–68 years). Patients visiting the ED during the study period were almost exclusively Asian (99.5%, excluding unknown), and a slight predominance of females (54.2%).

### 3.1. Transport

The majority of patients visiting the ED were self-referred (95.6%). Of these, 59.2% arrived by car or taxi; all other patients were pedestrians or arrived by bicycle. Only 95 patients arrived at the ED by ambulance.

### 3.2. Complaints

The four most common complaints were fever (*N* = 338), stomach and abdominal pain (*N* = 324), vertigo/dizziness (*N* = 236), and cough (*N* = 219; see Table 1). Prevalence of the other complaints ranged from four (diabetes-related) and eight (intoxication) to 164 (both headache and trauma). Of all patients, 532 patients visited the ED with multiple complaints. Of those reporting with only one complaint, the most frequent complaints included stomach and abdominal pain (*N* = 256), fever (*N* = 205), vertigo/dizziness (*N* = 199), chest pain (*N* = 140), and skin-related problems (*N* = 101).

### 3.3. Specialist

Overall, 41.3% of patients were referred to an internist (Table 1). Other frequently consulted physicians included a neurologist (13.7%), a pulmonologist (10.7%), a general surgeon (9.0%), and an orthopedic surgeon (8.6%). Of the 222 patients referred to a specialist that was not prespecified in the questionnaire, 104 were consulted by a dermatologist, 52 by an ENT specialist, and 23 by an ophthalmologist. During the study period no patients were referred to a psychiatrist or thoracic surgeon. 

For the six most consulted disciplines, Table 2 shows a detailed overview of the complaints for which patients are referred to them. The specialists to whom patients with only one complaint were referred are shown in Table 3. Patients with stomach or abdominal pain predominantly went to an internist or general surgeon. Nearly all patients (95.6%) with fever were referred to an internist. Out of the 199 patients with vertigo or dizziness, 179 visited the neurologist. Most of the patients with chest pain were seen by an internist. The fifth most common complaint, skin-related problems, was referred to a dermatologist in 88.1% of cases. 

## 4. Discussion

This study provides a demographic description of the patients who visited the ED of the Ruijin Hospital in Shanghai, China, and gives a better understanding of the organization and patient flow. 

Although emergency medicine is a separate discipline and is taught at the medical schools in China [6], a multidisciplinary model is still used within the Ruijin Hospital. The 2183 patients enrolled were referred to specialists of at least 12 different disciplines. The most frequently visited specialisms were internal medicine, neurology, pulmonology, general surgery, orthopedic surgery, and dermatology. The variety of complaints was very high. Only limited epidemiological information about the healthcare system in China is available. In general, the disease spectrum in China has evolved into a pattern that is similar to that of other developed countries, especially in large cities such as Shanghai [1]. The leading causes of death (2002 estimates) are cardiovascular disease (including cardiovascular attack), cancer, chronic obstructive pulmonary disease, and trauma [7]. This may be the consequence of a high prevalence of hypertension (due to high salt intake), air pollution, and traffic and industrial accidents [1]. Therefore, the high rates of patients reporting to the ED with upper airway complaints, pulmonary complaints, and musculoskeletal injuries as observed in our study were expected. Patients suffering from fever as sole complaint are all referred to an internist. This population represents approximately one third of the patients sent to this discipline. In total, 164 patients reported to the ED with headache, of which 86 as sole complaint. Almost 90% of these 86 patients were seen by a neurologist or neurosurgeon. However, 48 patients with headache and fever were first referred to an internist; these patients might have suffered from meningitis. We cannot deduce from our data if these patients had subsequently been sent to a neurologist. 

 There is no clear rationale for the relatively high prevalence of patients with skin-related problems. Most of these patients were referred to a dermatologist, indicating that these patients most likely did not suffer from traumatic skin lesions. Patients with burn wounds report directly to the Burn Center ED. Soft tissue injuries were probably treated by a general surgeon, since 48 patients with trauma were referred to a general instead of an orthopedic surgeon. A substantial number of patients reported complaints of vertigo/dizziness, for which no clear reason could be given. Most likely, this involved neurological disorders, as patients would be referred to an ENT specialist if the dizziness was due to vestibular organ disorder or to an internist if due to hypotension or anemia.

In Shanghai, unlike in other industrial countries, there is no general practitioner care system, where patients with minor acute or chronic disorders should be assessed first. The general practitioner can subsequently refer patients to the hospital for further treatment if necessary. As a consequence, physicians at the ED also treat a substantial number of patients with nonacute and nonsevere complaints. This is supported by the finding that only a small number of patients were transported to the hospital by ambulance (4.4%).

## 5. Conclusions

No previous demographic data of the patients and their indication for referral to a specialist of the Emergency Department in China were available. This study provides the first demographic description of patients visiting the ED of a large teaching hospital in Shanghai. Our results provide a better understanding of the reason for the ED visit and the triage system at the ED of the Ruijin Hospital in Shanghai. The results can be used in order to improve facilities appropriate for the specific population in the ED. Supplemental data on the diagnostics performed, the treatment given, and the outcome of the patients visiting the ED is needed to gain further insight into emergency medicine in the Ruijin Hospital.

## Figures and Tables

**Table 1:** Demographic description of patients reporting to the Triage Desk.

	Total population		Patients with one complaint	
	*N* = 2183	%	*N* = 1651	%
Gender				
Male	985	45.1	734	44.5
Female	1184	54.2	906	54.9
Unknown	14	0.6	11	0.7
Race				
Asian	2075	95.1	1559	94.4
Non-Asian	11	0.5	10	0.6
Unknown	97	4.4	82	5.0
Transport				
Ambulance	95	4.4	72	4.4
Self-referral	2088	95.6	1579	95.6
Complaints				
Headache	164	7.5	86	5.2
Vertigo/dizziness	236	10.8	199	12.1
Earache or ear infection	17	0.8	13	0.8
Throat symptoms	67	3.1	31	1.9
Chest pain	154	7.1	140	8.5
Shortness of breath	76	3.5	24	1.5
Cough	219	10.0	87	5.3
Stomach and abdominal pain	324	14.8	256	15.5
Urologic	128	5.9	74	4.5
Gynecologic symptoms	19	0.9	12	0.7
Spine	21	1.0	12	0.7
Upper extremity	55	2.5	18	1.1
Lower extremity	129	5.9	74	4.5
Skin	112	5.1	101	6.1
DM related	4	0.2	4	0.2
Fever	338	15.5	205	12.4
Trauma	164	7.5	72	4.4
Blood transfusion	39	1.8	34	2.1
Intoxication	8	0.4	5	0.3
Other	181	8.3	141	8.5
Unknown	90	4.1	63	3.8
Specialist				
Neurosurgeon	40	1.8	31	1.9
Neurologist	299	13.7	264	16.0
Ear-nose-throat specialist	52	2.4	42	2.5
Ophthalmologist	23	1.1	21	1.3
Dentist	16	0.7	14	0.8
Pulmonologist	239	10.9	106	6.4
General surgeon	197	9.0	154	9.3
Urologist	79	3.6	54	3.3
Obstetrist/gynecologist	25	1.1	18	1.1
Orthopedic surgeon	187	8.6	114	6.9
Internist	902	41.3	713	43.2
Dermatologist	104	4.8	96	5.8
Unknown	43	1.9	36	2.2

Data are shown as numbers with percentages. On the right-hand side, data are given for patients reporting to the ED with only one complaint.

**Table 2:** Frequencies of complaints for the six most commonly consulted disciplines.

Complaint	Internist	Neurologist	Pulmonologist	General surgeon	Orthopedic surgeon	Dermatologist
	*N* = 902	*N* = 299	*N* = 239	*N* = 197	*N* = 187	*N* = 104
Headache	60	93	3	0	0	0
Vertigo/dizziness	25	206	0	0	0	0
Ear ache or ear infection	12	0	0	0	0	0
Throat symptoms	35	0	2	1	0	0
Chest pain	149	0	4	1	0	0
Shortness of breath	14	1	60	0	0	0
Cough	21	0	197	0	0	0
Stomach and abdominal pain	179	0	1	108	1	0
Urologic	52	2	0	1	1	0
Gynecologic symptoms	1	0	0	0	0	1
Spine	0	0	0	2	19	0
Upper extremity	1	5	0	17	29	3
Lower extremity	3	2	0	16	110	0
Skin	3	0	0	1	5	95
DM related	4	0	0	0	0	0
Fever	293	6	22	5	0	2
Trauma	0	0	0	48	86	0
Blood transfusion	39	0	0	0	0	0
Intoxication	6	2	0	0	0	0
Other	100	6	5	9	3	4
Unknown	46	6	6	15	5	4

Data are shown for the entire study population.

**Table 3:** Frequencies of specialist referral for the five most common complaints.

Specialist	Stomach/abd. pain	Fever	Vertigo/dizziness	Chest pain	Skin
	*N* = 256	*N* = 205	*N* = 199	*N* = 140	*N* = 101
Neurosurgeon	0	0	1	0	0
Neurologist	0	3	179	0	0
Pulmonologist	1	4	0	1	0
General surgeon	94	1	0	1	0
Urologist	5	0	0	0	0
Obstetrist/gynecologist	7	0	0	0	0
Internist	148	196	18	138	0
Dermatologist	0	0	0	0	89
Unknown	1	1	1	0	12

Data are shown only for patients reporting to the ED with one complaint.
